# O-5S quantitative real-time PCR: a new diagnostic tool for laboratory confirmation of human onchocerciasis

**DOI:** 10.1186/s13071-017-2382-3

**Published:** 2017-10-02

**Authors:** Solomon A. Mekonnen, Marcus Beissner, Malkin Saar, Solomon Ali, Ahmed Zeynudin, Kassahun Tesfaye, Mulatu G. Adbaru, Florian Battke, Sven Poppert, Michael Hoelscher, Thomas Löscher, Gisela Bretzel, Karl-Heinz Herbinger

**Affiliations:** 1 Department of Medical Laboratory Sciences and Pathology, College of Health Sciences, Jimma University, Jimma, Ethiopia; 2 Department Microbial, Cellular and Molecular Biology, Addis Ababa University, Addis Ababa, Ethiopia; 3 Department of Infectious Diseases and Tropical Medicine, Medical Center of the University of Munich, Munich, Germany; 4 Dr. Battke SCIENTIA GmbH, Taufkirchen, Germany; 5 University Medical Center Hamburg-Eppendorf, University of Hamburg, Hamburg, Germany; 6 German Centre for Infection Research (DZIF), partner site Munich, Munich, Germany

**Keywords:** Confirmation, Microscopy, Molecular diagnosis, Onchocerciasis, O-5S qPCR, O-150 qPCR, Ethiopia

## Abstract

**Background:**

Onchocerciasis is a parasitic disease caused by the filarial nematode *Onchocerca volvulus.* In endemic areas, the diagnosis is commonly confirmed by microscopic examination of skin snip samples, though this technique is considered to have low sensitivity. The available melting-curve based quantitative real-time PCR (qPCR) using degenerated primers targeting the O-150 repeat of *O. volvulus* was considered insufficient for confirming the individual diagnosis, especially in elimination studies. This study aimed to improve detection of *O. volvulus* DNA in clinical samples through the development of a highly sensitive qPCR assay.

**Methods:**

A novel hydrolysis probe based qPCR assay was designed targeting the specific sequence of the *O. volvulus* O-5S rRNA gene. A total of 200 clinically suspected onchocerciasis cases were included from Goma district in South-west Ethiopia, from October 2012 through May 2013. Skin snip samples were collected and subjected to microscopy, O-150 qPCR, and the novel O-5S qPCR.

**Results:**

Among the 200 individuals, 133 patients tested positive (positivity rate of 66.5%) and 67 negative by O-5S qPCR, 74 tested positive by microscopy (37.0%) and 78 tested positive by O-150 qPCR (39.0%). Among the 133 O-5S qPCR positive individuals, microscopy and O-150 qPCR detected 55.6 and 59.4% patients, respectively, implying a higher sensitivity of O-5S qPCR than microscopy and O-150 qPCR. None of the 67 individuals who tested negative by O-5S qPCR tested positive by microscopy or O-150 qPCR, implying 100% specificity of the newly designed O-5S qPCR assay.

**Conclusions:**

The novel O-5S qPCR assay is more sensitive than both microscopic examination and the existing O-150 qPCR for the detection of *O. volvulus* from skin snip samples. The newly designed assay is an important step towards appropriate individual diagnosis and control of onchocerciasis.

**Electronic supplementary material:**

The online version of this article (10.1186/s13071-017-2382-3) contains supplementary material, which is available to authorized users.

## Background

Onchocerciasis, also referred to as “river blindness,” is caused by the filarial nematode *Onchocerca volvulus*, which is transmitted to humans by black flies (*Simulium* spp.) in endemic countries of Africa, including Ethiopia, three countries in Latin America, and in Yemen. This parasitic disease was listed by the World Health Organization (WHO) as one of the eighteen neglected tropical diseases [1–4]. Onchocerciasis constitutes a major public health problem in affected regions, as an estimated 37 million persons are infected with *O. volvulus* worldwide, with the vast majority of them living in East and West Africa. As this disease is endemic in some of the world’s poorest areas, it had a major impact on the economic and social aspects of affected communities [5–7]. Ethiopia is one of the countries with a high burden of onchocerciasis, where the disease has spread widely to regions that were previously non-endemic [8]. In 1998, the “African Program for Onchocerciasis Control” sponsored the first nationwide epidemiological mapping of onchocerciasis in Ethiopia. Following the results of that study, most areas of Central, South, and West Ethiopia were defined endemic [9].

To avoid severe progression of the disease, early detection and treatment are essential. A clinically suspected *O. volvulus* infection can be confirmed by different laboratory methods: microscopic detection of microfilariae (mf) from skin snip samples (SSS) following incubation in normal saline; detection of the major sperm protein 2 (MSP2) by dipstick test; detection of recombinant antigens by means of a luciferase immune precipitation system (QLIPS); and rapid DNA detection test strips; and polymerase chain reaction (PCR) [10–12]. The microscopic examination is cheap and easy to perform. However, this method has several limitations, as its sensitivity depends on the location where skin snips were taken, mf density, stage of disease, previous treatment, and especially on the qualifications of staff performing tests [13–16].

Various PCR-based assays were described for the detection of different larval stages of *O. volvulus* [17–22]. For more than a decade, a conventional gel-based PCR applying degenerated primers amplifying the *O. volvulus*-specific O-150 tandem repeat [23–25] was often used to confirm onchocerciasis from SSS [14, 26, 27]. In 2011, Fink et al. described a melting curve based quantitative real-time PCR (qPCR) which also used degenerated primers to detect a 154 bp amplicon of the O-150 repeat (O-150 qPCR) [28]. However, the long PCR amplicon may lead to limited sensitivity, and the lack of a hybridization probe may reduce the specificity of this assay [29]. In recent years, Lloyd et al. [14] and Golden et al. [30] showed the importance of PCR for the detection of *O. volvulus*.

In Ethiopia, cases of onchocerciasis are routinely diagnosed by microscopic examination of SSS, which has limited sensitivity especially in samples with low mf density and is therefore not sufficiently reliable for programmatic monitoring and evaluation of the effects of the WHO recommended mass drug administration [31]. The objective of this study was to develop and validate a novel hydrolysis probe-based qPCR targeting the 5S rRNA gene of *O. volvulus* (O-5S qPCR) with enhanced sensitivity for the detection of *O. volvulus* DNA from SSS of clinically diagnosed patients from Ethiopia. The diagnostic validity of the novel qPCR assay was compared with direct microscopy and the established O-150 qPCR. The study furthermore compared socio-demographic data, potential risk factors for onchocerciasis, clinical symptoms and medical histories with the laboratory results.

## Methods

### Study area and inclusion criteria

The study was conducted in Goma district, which has a population of approximately 213,000 inhabitants. Goma is one of the 13 districts in Jimma zone, situated in Oromia region in Ethiopia [32]. Goma district has 36 rural and three urban “kebeles” (i.e. smallest administrative unit in Ethiopia). The number of study participants was calculated in proportion to the total number of inhabitants in each of these kebeles. The following numbers of study participants were included from four randomly chosen kebeles: Dedessa (*n* = 28), Omo Gobu (*n* = 52), Belfo Konche (*n* = 84) and Kilole Qirqir (*n* = 36). Individuals living in the study area with any form of the following clinical manifestation(s) were included in the present study: papular rash, scarring “leopard skin” (i.e. spotted depigmentation of the skin) and skin itching around the buttocks. Patients who received ivermectin treatment during the past six months were excluded.

### Study design and data collection

In this cross-sectional study, data from 200 clinically suspected onchocerciasis individuals were collected from October 2012 through May 2013. After written informed consent was obtained, study participants were interviewed by trained study nurses to record socio-demographic data, potential risk factors for onchocerciasis and medical history by pre-designed and pre-tested questionnaires. Data were completely pseudonymized and transferred to an Excel-based database (Microsoft, Redmond, WA, USA). SSS were collected from either side of the buttocks by senior medical laboratory technologists according to routine standardized procedures [1].

### Microscopy

The collected SSS was transferred to one cavity of a 96-well plate, leaving blank cavities between samples to avoid cross contamination. Three drops of phosphate buffered saline (PBS, Sigma-Aldrich, Bangalore, India) were added to allow complete release of mf during a four h incubation period. The samples were sealed by Titer Tops sealing film for microplates (Sigma-Aldrich) and transported to a laboratory of health centers nearby the study areas for wet mount microscopic examination. There, PBS from SSS was transferred to microscopic slides for the detection of *O. volvulus* mf with light microscopy under 10- and 40-fold objectives by experienced senior laboratory technologists*.* The remaining samples were stored in the respective 96-well plates, transported within 24 h to the Department of Medical Laboratory Sciences and Pathology of Jimma University, and there immediately deep-frozen at -20 °C until shipment by courier service to the Department for Infectious Diseases and Tropical Medicine (DITM) of the Ludwig-Maximilians University in Munich, Germany.

### O-5S qPCR assay

#### Target sequence and primer design

A qPCR assay was developed for the detection of *O. volvulus* from human SSS. After an extensive database search (GenBank [PubMed, NCBI]) and sequence analyses (DNASIS Max software [MiraiBio, San Francisco, CA]), an intergenic spacer region of the *O. volvulus* 5S rRNA gene was chosen as a target for PCR amplification. Primers and a hydrolysis probe (MWG Eurofins, Ebersberg, Germany) for amplification of this *O. volvulus-*specific intergenic spacer region of the 5S rRNA gene were designed by alignment of 5S rRNA gene sequences (DNASIS Max) retrieved from GenBank [33] from closely related filarial nematodes potentially contaminating the SSS from human skin by primer 3. Specificity of the primers for *O. volvulus* was confirmed in silico using the basic local alignment search tool (BLAST, GenBank, NCBI). The selected 5S rRNA target, primers and hydrolysis probe, and alignments of the target region from *O. volvulus* with *Wuchereria bancrofti*, *Brugyia malayi*, *Loa loa*, *Mansonella streptocerca* and *Plasmodium falciparum* are provided in Additional file 1.

#### O-5S qPCR standard

A PCR standard template was generated by gene synthesis of the target sequence and subsequent cloning to the pEX-A2 vector (MWG, Ebersberg, Germany). The vector was transformed into *E. coli* (JM109, Zymo, Freiburg, Germany) and the transformation mix was plated on LB agar plates with 100 mg/l ampicillin (Carl Roth, Karlsruhe, Germany) and cultivated at 37 °C overnight. By selection of one suitable colony, a master cryo culture was obtained and stored in RotiStore tubes (Carl Roth). From this master cryo culture, a 3 ml LB liquid culture with 100 mg/l ampicillin (Carl Roth) was grown over night. The plasmid DNA was extracted with a commercial plasmid extraction kit, according to the manufacturer’s instruction (HiYield Plasmid Mini kit; Süd-Laborbedarf, Gauting, Germany). The cloned plasmid sequence was confirmed by direct DNA sequencing as previously described [34]. The purity of extracted plasmid DNA was assessed by photometry on a BioPhotometer plus (Eppendorf, Wesseling-Berzdorf, Germany) and agarose gel-electrophoresis on a 1% TAE gel. The plasmid solution was quantified by a dsDNA fluorescence quantification kit (Qubit, Life Technologies, Karlsruhe, Germany), according to the manufacturer’s instruction. The number of fragments per μl was calculated and aliquots of the plasmid solution, diluted to the desired fragment numbers, were used for determination of the lower limit of detection (LOD, i.e. lowest template concentration rendering positive amplification of 95% of samples) [29].

#### O-5S qPCR protocol

The primers for O-5S qPCR were as described in Table 1. The reaction mixture (total volume: 20 μl) contained 8.6 μl molecular grade H_2_O (Carl Roth), 4 μl 5-fold PCR buffer qPCR Mix Plus (Solis BioDyne, Tartu, Estonia), 2 μl 10-fold Exo IPC Mix (TaqMan exogenous internal positive control reagent; ThermoFisher Scientific, Darmstadt, Germany), 1 μl of each primer and probe (10 μM), 0.4 μl 50-fold Exo IPC DNA (ThermoFisher Scientific) and 2 μl DNA template. The amplification was performed at 95 °C for 15 min, followed by 45 cycles of 95 °C for 15 s, 56 °C for 20 s and detection at 72 °C for 20 s on a Bio-Rad CFX-96 real-time PCR machine (Bio-Rad, Munich, Germany) and included negative extraction, negative “no template”, positive run controls as well as the internal positive controls (IPC).

**Table 1:** Sequences and nucleotide positions of primers and the hydrolysis probe, including the corresponding amplicon sizes of the O-5S qPCR

Test	Primer/probe^a^	Sequence (5'-3')^b^	Nucleotide position^c^	Amplicon size (bp)
O-5S qPCR	Ov5S-F	GAGGTAATTGAATGTTTCTGCCC	74–96	84
	Ov5S-R	*TGTTGTCCCGCTCATGC*	158–142	
	Ov5S-P	FAM-*AGTTTCGACTGCTGTGGCTTGAAGCG*-BHQ1	99–124	

^a^F, forward primer; R, reverse primer; P, hydrolysis probe (TibMolBiol, Berlin, Germany)

^b^Hydrolysis probe with Fluorescein (FAM) and BlackHole Dark Quencher (BHQ)

^c^Nucleotide positions are provided for the intergenic spacer region of the 5S ribosomal RNA gene of *Onchocerca volvulus* (GenBank: U31643.1)

#### Specificity and sensitivity of O-5S qPCR

Specificity of the assay was assessed by using 20 O-150 PCR positive (“must detect”) and 20 O-150 PCR negative (“must not detect”) *O. volvulus* clinical DNA extracts from SSS collected in a previous study [35], confirmed DNA extracts from *Dirofilaria immitis* (*n* = 3), *D. repens* (*n* = 1), *Loa loa* (*n* = 1) and *Mansonella streptocerca* (*n* = 1). The analytical sensitivity of the O-5S qPCR assay was determined as LOD by using 10-fold serial dilutions of O-5S plasmid standards with known copy numbers. Intra- and inter-assay variability were assessed by testing each sample in quadruplicate within one 96-well plate, repeated on three different days. Variability was judged low if the maximum cycle threshold variation range (C_q_-range_max_; i.e. range of C_q_-values of samples tested in the same dilution) was ≤0.5 (intra-assay) and ≤1.0 (inter-assay).

### O-150 melting curve based qPCR

The melting curve based O-150 qPCR assay was conducted according to the protocol described by Fink et al. [28] on a Bio-Rad CFX-96 real-time PCR machine (Bio-Rad) and included negative extraction, negative “no template” and positive run controls.

### O-150 hybridization probe based qPCR assay

The O-150 hybridization probe based qPCR assay was performed as described by Golden et al. 2016 [30] with minor modifications as the original qPCR protocol did not lead to any amplicons. Briefly, the sequences of primers and probe were: OvFWD 5′-TGT GGA AAT TCA CCT AAA TAT G-3′, OvREV 5′-AAT AAC TGA TGA CCT ATG ACC-3′ (Eurofins Genomics, Ebersberg, Germany), and OvProbe 5′-6FAM-TAG GAC CCA ATT CGA ATG TAT GTA CCC-MGBNFQ-3′ (minor groove binding TaqMan® Probe #5208995 P/N 4316033, Applied Biosystems, Inchinnan, UK). The qPCR assay was designed to amplify the O-150 repeat of O. volvulus using 7.5 μl SuperHot Master Mix (2×) (BIORON, Ludwigshafen, Germany), 0.1 μl of 100 μM forward primer, 0.1 μl of 100 μM reverse primer, 0.1 μl of 100 μM probe, and 7.2 μl DNA extract/negative control (nuclease free water), positive control (O150 plasmid standard). Finally, the O-150 qPCR was run in a total volume of 15 μl with an initial denaturation at 94 °C for 2 min, [denaturation at 94 °C for 15 s, hybridization at 49 °C for 30 s, and elongation at 60 °C for 2 min] × 45 cycles. All test or control samples were run in a single well and analyzed using an Illumina Eco Real-time PCR system. An O-150 plasmid standard (Genbank accession number J04659.1) was generated by gene synthesis (Eurofins Genomics). The maximum quantification cycle for a positive result was set to 37 according to the lowest detectable plasmid standard concentration. This assay was performed in an observer-blinded way without knowledge of microscopy or other PCR results.

### Comparison of O-150 and O-5S qPCR assays

#### Sample transport

The SSS were thawed and transferred into 2 ml screw cap tubes (Sarstedt, Nümbrecht, Germany) containing 700 μl cell lysis buffer (CLS, Qiagen, Hilden, Germany) to allow transport at ambient temperature without DNA degradation by courier service to DITM.

#### DNA extraction

The dsDNA of *O. volvulus* was extracted using the Puregene DNA extraction tissue kit (Qiagen) using enzymatic lysis as described elsewhere [35] at DITM. Briefly, SSS were inactivated at 95 °C for 15 min. Subsequently, samples were incubated overnight at 55 °C in 700 μl CLS enriched with proteinase K (Sigma-Aldrich) to a final concentration of 300 μg/ml. The proteinase K was inactivated at 95 °C for 15 min. After the specimens were cooled to ambient temperature, lysozyme (Qiagen) was added to a final concentration of 250 μg/ml and specimens were incubated at 37 °C for one h. Finally, the DNA pellets were resuspended in 200 μl DNA hydration solution (Qiagen). DNA extracts were stored at 4 °C (short-term, up to one week) or -20 °C (long-term storage) until further procession by PCR.

#### qPCR assays

At DITM DNA extracts from SSS were subjected to O-150 qPCR and to the novel O-5S qPCR assays by independent and blinded laboratory technicians who were unaware of the microscopic results.

#### Statistical analyses

All data were stored in a Microsoft Excel database (Microsoft). Bivariate tests (chi-square tests including Fisher’s exact test and McNemar chi-square test for matched pairs of samples with categorical test results), multivariate logistic regression, t-tests as parametric test, and estimation of standard error of proportion (to calculate 95% confidence intervals, 95% CI, of categorical test results) were conducted using EpiInfo, version 3.3.2. (Centers for Disease Control and Prevention, Atlanta, GA) and Stata software, version 9.0. (Stata Corporation, College Station, TX). Significant differences were defined as *p* values below 0.05 or as non-overlapping 95% CI of proportions. The sensitivity and specificity of microscopy and O-150 qPCR were calculated by comparing these results with those of O-5S qPCR as a reference test.

## Results

### Novel O-5S qPCR results

Out of 266 individuals with clinically suspected onchocerciasis, 200 (75.19%) fulfilled the inclusion criteria and composed the study population. Among these, 133 (66.50%) tested positive by the newly developed O-5S qPCR assay. Interestingly, none of the individuals who tested negative by the novel O-5S qPCR tested positive by microscopy or by O-150 qPCR assay (Table 2).

**Table 2:** Comparison of microscopy, O-150 qPCR assay and O-5S qPCR assay for the diagnosis of onchocerciasis

Variable		Study population *n* (%)	O-5S qPCR		*P*-value^a^
			Positive cases*n* (%)	Negative controls*n* (%)	
Sample size	Patients	200 (100)	133 (100)	67 (100)	na
Microscopy	Positive	74 (37.00)	74 (55.64)	0 (0)	< 0.01^a^*
	Negative	126 (63.00)	59 (44.36)	67 (100)	
O-150 qPCR	Positive	79 (39.50)	79 (59.40)	0 (0)	< 0.01^a^*
	Negative	121 (60.50)	54 (40.60)	67 (100)	
Microscopy + O-150 qPCR	Negative + negative	109 (54.50)	42 (31.58)	67 (100)	na
	Negative + positive	17 (8.50)	17 (12.78)	0 (0)	na
	Positive + negative	12 (6.00)	12 (9.02)	0 (0)	na
	Positive + positive	62 (31.00)	62 (46.62)	0 (0)	na

^a^McNemar’s chi-square test for matched pairs: *χ* ^2^ = 59.00 (microscopy and O-5S qPCR); *χ* ^2^ = 54.00 (O-150 qPCR and O-5S qPCR)

*Significant differences were defined as *P*-values < 0.05

*Abbreviation*: *na* not applicable

### Microscopy results

Among the 200 individuals comprising the study population, microscopic examination of SSS detected *O. volvulus* mf in 74 individuals, corresponding to a positivity rate of 37.00%. These individuals tested positive by the newly developed assay, O-5S qPCR, which corresponds to a sensitivity of 55.64% (95% CI: 47.20–64.08%), and none of the individuals who tested positive by microscopy tested negative by O-5S qPCR, which corresponds to a specificity of 100%. The probability for positive microscopy results to be true positive (positive predictive value) was 100%, and for negative results to be true negative (negative predictive value) was 53.17% (95% CI: 44.46–61.89) (Table 2).

### O-150 qPCR results

Among the 200 individuals clinically suspected for onchocerciasis, the O-150 qPCR assay by Fink et al. detected DNA of *O. volvulus* in 78 patients, which corresponds to a positivity rate of 39.00%. All these patients tested positive by O-5S qPCR, which corresponds to a sensitivity of 59.40% (95% CI: 50.28–67.02%), with none of these individuals testing negative by O-5S qPCR, which corresponds to a specificity of 100%. The probability for positive O-150 qPCR results to be true positive (positive predictive value) was 100%, and for negative results to be true negative (negative predictive value) was 54.92% (95% CI: 46.09–63.75) (Table 2).

From the total of 200 SSS collected among individuals clinically suspected for onchocerciasis, the O-150 qPCR assay by Golden et al. detected DNA of *O. volvulus* in 33 of them, which corresponds to a positivity rate of 16.5%. Of these 33 individuals, only 23 individuals tested positive by microscopy, the O-150 qPCR assay by Fink et al. assay and our newly developed O-5S qPCR assay, respectively. Unexpectedly, ten individuals that tested positive in the assay by Golden et al. tested negative with all other three diagnostic methods (Additional file 2: Table S1). Therefore, these results could not be used as reference standard.

### Performance characteristics of the novel O-5S qPCR assay

**Fig. 1:**
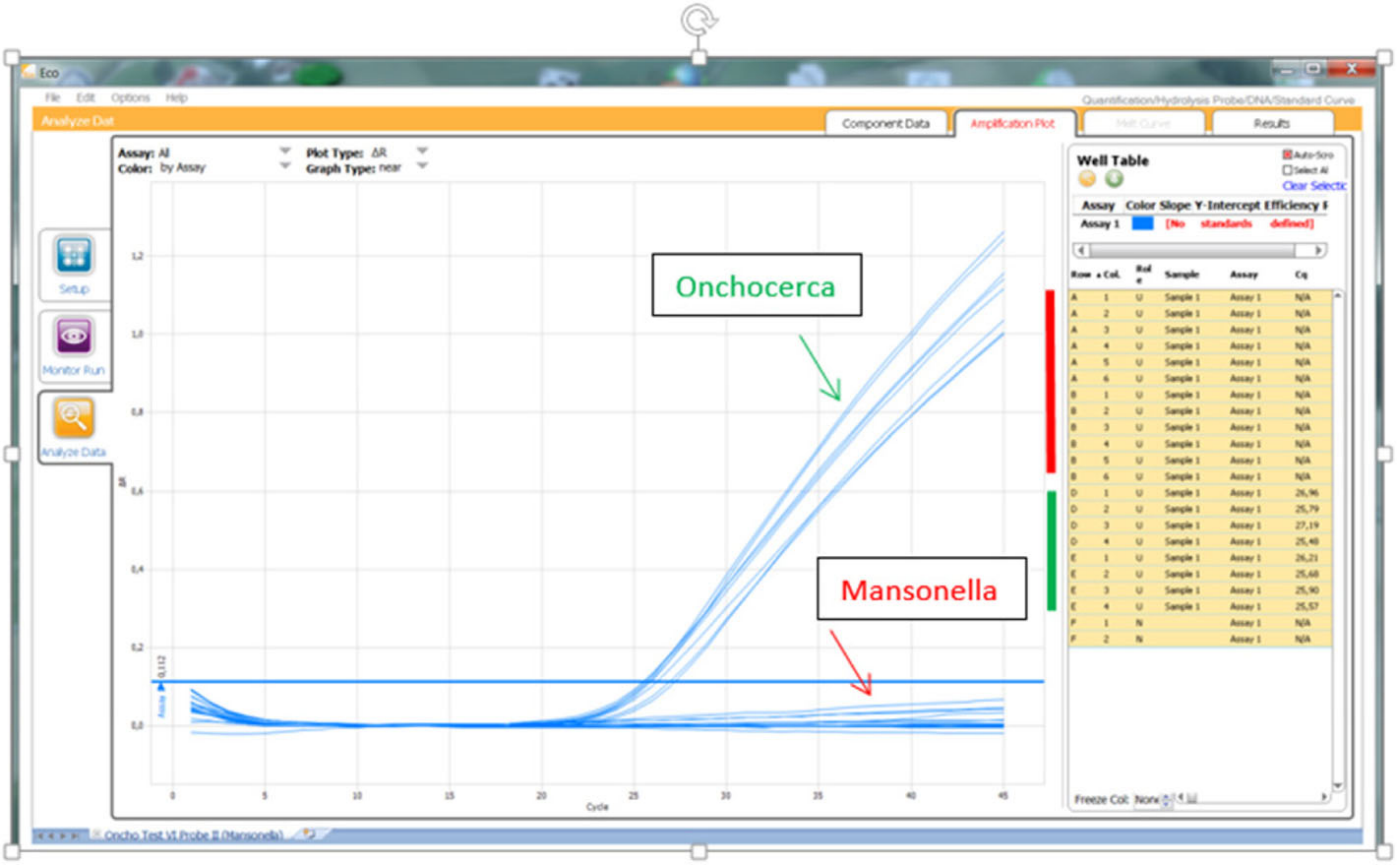
Assessment of the specificity of novel O-5S qPCR assay in the detection of *Onchocercia volvulus and Mansonella streptocerca* DNA

In silico analysis of the novel primers/probe revealed 100% specificity for *O. volvulus* among human pathogenic filariae, which excluded amplification of human DNA. Testing of “must detect O-5S samples” revealed positive amplification results for 20/20 (100%) *O. volvulus* DNA extracts, while all other “must not detect O-5S samples” remained negative. Therefore, the specificity of the novel O-5S qPCR was 100%. The LOD was six copies of the target sequence of *O. volvulus*. Intra- and inter-assay variabilities were low. The calibration curve is provided in Additional file 3: Figure S1. Furthermore, the novel O-5S qPCR assay does not amplify the DNA of *Mansonella streptocerca,* a closely related filarial parasite (Fig. 1).

### Concordance rate

Among the 133 individuals tested positive by O-5S qPCR, 61 (45.86%) had a positive microscopy result. Out of the 78 individuals tested positive by O-150 qPCR, 13 individuals (9.77%) had a positive microscopy result, 17 individuals (12.78%) had a positive result only with O-150 qPCR, and 42 (31.58%) had a negative result with both tests. The concordance rate between microscopy and O-150 qPCR was 77.44% (103/133) among individuals tested positive by O-5S qPCR. Among all 67 individuals tested negative by O-5S qPCRs, both tests resulted in negative results, corresponding to a concordance rate between microscopy and O-150 qPCR of 100% (67/67). In the study population of 200 patients, the overall concordance rate between microscopy and O-150 qPCR was 85% (170/200). Between microscopy and O-5S qPCR, the concordance rate was 70.5% (141/200), and between O-150 qPCR and O-5S qPCR, 72.5% (145/200) (Table 2). Of note, there is no correlation between both assays (Additional file 4: Figure S2).

### Diagnostic yield

The introduction of the O-150 qPCR to the already widely performed microscopy for the diagnosis of onchocerciasis has been shown in this study to provide an additional diagnostic yield of 13% (17/133 cases). By replacing microscopy with O-150 qPCR, the additional diagnostic yield would have been 3% (4/133 cases) in this study. Among the 133 individuals tested positive by O-5S qPCR, 91 (68.42%) tested positive by microscopy or O-150 qPCR. By performing the novel O-5S qPCR, an additional diagnostic yield of 42 (31.58%) was achieved (Table 2).

### Socio-demographics

Among the individuals who tested positive by the newly designed O-5S qPCR assay, the proportion of females (22.56%) was not significantly (*χ* ^*2*^ = 0.07, *df* = 1, *P* = 0.79) different from those tested negative (20.90%). The median age was 32 years among individuals who tested positive, whereas it was 15 years among those tested negative. The proportion of age group 30–72 years among those who tested positive (56.39%) was significantly (*χ* ^*2*^ = 9.85, *df* = 1, *P* < 0.01) higher than among those who tested negative (32.84%). The proportion of farmers among individuals tested positive (70.68%) was significantly (*χ* ^*2*^ = 22.14, *df* = 2, *P* < 0.01) higher than among those tested negative (37.31%), however, this association was highly confounded by age (Table 3).

**Table 3:** Socio-demographic characteristics of clinically suspected onchocerciasis subjects along with O-5S qPCR assay

Variable		Study population*n* (%)	Positive*n* (%)	Negative*n* (%)	*P*-value^a,b^
Sample size	Patients	200 (100)	133 (100)	67 (100)	na
Gender	Female	44 (22.00)	30 (22.56)	14 (20.90)	0.79^a^
	Male	156 (78.00)	103 (77.44)	53 (79.10)	
Age (years)	Range	3–72	3–72	5–66	na
	Median	29	32	15	na
	Interquartile range	12.75–45.00	20–45	10.0–35.5	na
	Age group: 3–29	103 (51.50)	58 (43.61)	45 (67.16)	< 0.01^a^*
	Age group: 30–72	97 (48.50)	75 (56.39)	22 (32.84)	
Occupation	Child, student	72 (36.00)	33 (24.81)	39 (58.21)	< 0.01^a^*
	Farmer	119 (59.50)	94 (70.68)	25 (37.31)	< 0.01^a^*
	Other occupation	9 (4.50)	6 (4.51)	3 (4.48)	1.00^b^

^a^Chi-square tests

^b^Fisher exact chi-square test

*Significant differences were defined as *P*-value < 0.05

*Abbreviation*: *na* not applicable

### Risk factors and symptoms

For the variables “distance of residence from river” (< 1 km: 84.96% / 74.63%; > 1 km: 15.04% / 25.37%) and “fetching water from river nearby residence” (Yes: 96.24% / 94.03%), no significantly different proportions were found among individuals tested positive and negative. For the variable “collecting wood from nearby forest” (Yes: 72.93% / 55.22%), the proportion were significantly (*χ* ^*2*^ = 6.29, *df* = 1, *P* = 0.01) higher among individuals tested positive than among those who tested negative. Also for the variables “palpable inguinal lymph nodes” (43.61% / 20.90%) and “skin itching around buttock” (74.44% / 46.27%), the proportions were significantly (*χ* ^*2*^ = 9.77, *df* = 1, and *χ* ^*2*^ = 15.46, *df* = 1, respectively, *P* < 0.01 each) higher among individuals who tested positive than those who tested negative (Table 4).

**Table 4:** Assessment of potential risk factors for onchocerciasis through O-5S qPCR assay

Variable		Study population *n* (%)	Positive *n* (%)	Negative *n* (%)	*P*-value^a,b^
Sample size	Patients	200 (100)	133 (100)	67 (100)	na
Distance of residence from river	< 1 km	163 (81.50)	113 (84.96)	50 (74.63)	0.08^a^
	> 1 km	37 (18.50)	20 (15.04)	17 (25.37)	
Fetching water from river nearby residence	Yes	191 (95.50)	128 (96.24)	63 (94.03)	0.49^b^
	No	9 (4.50)	5 (3.76)	4 (5.97)	
Collecting wood from nearby forest	Yes	134 (67.00)	97 (72.93)	37 (55.22)	0.01^a^*
	No	66 (33.00)	36 (27.07)	30 (44.78)	
Palpable inguinal lymph nodes	Not known	2 (1.00)	1 (0.75)	1 (1.49)	na
	Yes	72 (36.00)	58 (43.61)	14 (20.90)	< 0.01^a^*
	No	126 (63.00)	74 (55.64)	52 (77.61)	
Skin itching around buttock	Yes	130 (65.00)	99 (74.44)	31 (46.27)	< 0.01^a^*
	No	70 (35.00)	34 (25.56)	36 (53.73)	

^a^Chi-square tests

^b^Fisher exact chi-square test

*Significant differences were defined as *P*-value < 0.05

*Abbreviation*: *na* not applicable

Multivariate logistic regression has shown association only for age and itching of the skin with positive results of O-5S qPCR. Among those who tested positive, individuals were more likely 30 years or older than among individuals tested negative (adjusted OR = 2.17; 95% CI: 1.11–4.23). Among those who tested positive, individuals reported itching around buttocks more often than individuals tested negative (adjusted OR = 2.13; 95% CI: 1.04–4.47) (Table 4).

## Discussion

This is the first study assessing a novel O-5S quantitative real-time PCR for the molecular confirmation of *O. volvulus* infections in Goma district, Southwest Ethiopia, which is an endemic region for onchocerciasis. The present study showed that the newly developed assay is more sensitive than both microscopic examination of SSS and the conventional O-150 qPCR assay for the diagnosis of human onchocerciasis. This might be attributable to the implementation of a very stable hydrolysis probe that is 100% specific for *O. volvulus* from clinical samples*.* Moreover, the data presented in this study showed that the O-5S qPCR assay for the diagnosis of onchocerciasis from SSS can detect low-density DNA of *O. volvulus*. This is especially needed for reliable programmatic monitoring and evaluations regarding national elimination campaigns [31]. Furthermore, the novel assay was highly sensitive, approaching a lower limit of detection of six templates of the target sequence, which increased the detection limit by half in comparison to the O-150 qPCR [28]. This might be due to the careful choice of the target sequence with only moderate secondary structure forming primers, which prevent the formation of primer-primer dimers [29].

The results of the present study did not show any significant differences between microscopy and O-150 qPCR. These two tests had comparable positivity rates of 37% and 39%, respectively. Furthermore, the concordance rate between microscopy and O-150 qPCR was high: 77% among individuals tested positive by O-5S qPCR, 100% among individuals tested negative by O-5S qPCR, and 85% of all clinically suspected patients. These results indicate that the additional introduction of the O-150 qPCR would not substantially increase the diagnostic yield (13%). Completely replacing microscopy by O-150 qPCR would have even decreased the diagnostic yield by 3% in this study. In contrast, application of the novel O-5S qPCR resulted in an additional diagnostic yield of 32%. The O-5S qPCR had a much higher sensitivity in comparison to microscopy (56%) or O-150 qPCR (59%), whereas the two conventional tests had limited concordance rates of 71% and 73%, respectively, compared to O-5S qPCR. Although the sensitivity of the two conventional tests is limited, the present study showed that both tests reached a specificity of 100% for *O. volvulus.*

Previously, different studies were conducted in various regions of Ethiopia to determine the prevalence of onchocerciasis using direct microscopy from SSS. In the present study, the positivity rate for microscopic detection of onchocerciasis was 37%, a finding comparable to positivity rates as described in other studies from Ethiopia 31% in Bebeka [36] and 34% in Blue Nile valley [37]. On the other hand, the positivity rate of onchocerciasis in the present study was higher than reports from other locations in Ethiopia 17% Gilgel Ghibe river valley [38], 23% Kaso Hixi [39] and 22% in Teppi area [40] and lower than a study conducted in Anfilo district (75%) [1]. Of note, our finding has initiated the Zonal health bureau to launch ivermectin mass treatment which benefits not only the study participant but also other individuals living in the study area which was not included in the present study.

Among individuals tested positive by O-5S qPCR, the prevalence of males (77%) was higher than that of females (23%). This finding is in line with previous studies that reported a higher proportion of males among patients with onchocerciasis [1, 33], which could be due to occupational exposure and susceptibility of study participants. In the rural parts of Ethiopia, males are more involved in outdoor activities than females and prefer to wear short pants while performing outdoor activities [1]. Thus, outdoor activities and wearing short pants may render males more prone to black fly’s bites.

## Conclusions

The newly designed assay for the detection of *O. volvulus* DNA from SSS shows an improvement in performance and an important step towards appropriate diagnosis of onchocerciasis. This, in turn, can provide a crucial contribution to disease progress monitoring including reliable programmatic monitoring and evaluation of MDA, for example, to control the elimination of onchocerciasis. Consequently, future evaluation and demonstration trials are planned to apply the novel O-5S qPCR in endemic regions of Ethiopia. Moreover, the novel O-5S qPCR could serve as a valuable tool for future drug trials, especially if detection and quantification of low mf densities from tissue samples are required.

## Additional files

Additional file 1: Text. Selection of *O. volvulus* 5S sequence and alignment of target sequence from *O. volvulus* with related nematode sequences and *Plasmodium falciparum. (DOCX 24 kb)*

Additional file 2: Table S1. Additional file 2: Table S1. Socio-demographic, clinical and diagnostic data of the study subjects. (XLSX 224 kb)

Additional file 3: Figure S1. Calibration curve of the novel O5-S qPCR. (DOCX 22 kb)

Additional file 4: Figure S2. XY-graph showing the correlation between O-150 qPCR and O-5S qPCR. (DOCX 19 kb)

## Abbreviations

DITM: Department for Infectious Diseases and Tropical Medicine
ME: minimum evolution
mf: microfilariae
SSS: skin snip sample
