# Supplementary material for: O-5S quantitative real-time PCR: a new diagnostic tool for laboratory confirmation of human onchocerciasis
Source: Parasit Vectors. 2017 Oct 2;10:451. doi: 10.1186/s13071-017-2382-3 (PMC5625774; doi:10.1186/s13071-017-2382-3)
Supplement: Supplementary file 1 — Selection of O. volvulus 5S sequence and alignment of target sequence from O. volvulus with related nematode sequences and Plasmodium falciparum. (DOCX 24 kb) [file 13071_2017_2382_MOESM1_ESM.docx]

**Additional file 1-** Selection of O. volvulus 5S sequence and alignment of target sequence from *O. volvulus* with related nematode sequences and *Plasmodium falciparum*

With Primer-BLAST (NCBI) the following part of the sequence was determined as real-time PCR amplicon for specific detection of *O. volvulus (primer binding site highlighted in gray, probe binding site highlighted in yellow)*:

***GAGGTAATTGAATGTTTCTGCCC***AGAGTTTCGACTGCTGTGGCTTGAAGCGAAATTTTGGAACGTCCT***GC***

***ATGAGCGGGACAACA***

Primer sequence forward: GAG GTA ATT GAA TGT TTC TGC CC

Primer sequence reverse: TGT TGT CCC GCT CAT GC

Probe sequence: **FAM**-AGT TTC GAC TGC TGT GGC TTG AAG CG-**BHQ1**

LOCUS OVU31643 159 bp DNA linear INV 12-MAY-1997

DEFINITION Onchocerca volvulus 5S ribosomal RNA intergenic spacer region,

partial sequence.

ACCESSION U31643

VERSION U31643.1 GI:975836

KEYWORDS .

SOURCE Onchocerca volvulus

ORGANISM [Onchocerca volvulus](http://www.ncbi.nlm.nih.gov/Taxonomy/Browser/wwwtax.cgi?id=6282)

Eukaryota; Metazoa; Ecdysozoa; Nematoda; Chromadorea; Spirurida;

Filarioidea; Onchocercidae; Onchocerca.

REFERENCE 1 (bases 1 to 159)

AUTHORS Xie,H., Bain,O. and Williams,S.A.

TITLE Molecular phylogenetic studies on filarial parasites based on 5S

ribosomal spacer sequences

JOURNAL Parasite 1 (2), 141-151 (1994)

PUBMED [9140481](http://www.ncbi.nlm.nih.gov/pubmed/9140481)

REFERENCE 2 (bases 1 to 159)

AUTHORS Williams,S.A.

TITLE Direct Submission

JOURNAL Submitted (14-JUL-1995) Steven A. Williams, Biological Sciences,

Smith College, Clark Science Center, Northampton, MA 01063, USA

FEATURES Location/Qualifiers

source 1..159

/organism="Onchocerca volvulus"

/mol_type="genomic DNA"

/db_xref="taxon:[6282](http://www.ncbi.nlm.nih.gov/Taxonomy/Browser/wwwtax.cgi?id=6282)"

[misc_RNA](http://www.ncbi.nlm.nih.gov/nuccore/975836?from=1&to=159&sat=4&sat_key=34389703) <1..>159

/note="5S ribosomal RNA intergenic spacer region"

ORIGIN

1 taattatttt tgaatgtaca acagtcatat gagaatgaag tagttacaaa cattggttta

61 attacccaag tttgaggtaa ttgaatgttt ctgcccagag tttcgactgc tgtggcttga

121 agcgaaattt tggaacgtcc tgcatgagcg ggacaacaa

**Alignment of target sequence from *O. volvulus* with *Loa Loa***

Loa loa isolate G9 5S ribosomal RNA gene region

Sequence ID: [KR080179.1](https://www.ncbi.nlm.nih.gov/nucleotide/929558944?report=genbank&log$=nuclalign&blast_rank=26&RID=DURAUWV4016)Length: 362Number of Matches: 1

Related Information

Range 1: 209 to 295[GenBank](https://www.ncbi.nlm.nih.gov/nucleotide/929558944?report=genbank&log$=nuclalign&blast_rank=26&RID=DURAUWV4016&from=209&to=295)[Graphics](https://www.ncbi.nlm.nih.gov/nuccore/929558944?report=graph&rid=DURAUWV4016%5b929558944%5d&tracks=%5bkey:sequence_track,name:Sequence,display_name:Sequence,id:STD1,category:Sequence,annots:Sequence,ShowLabel:true%5d%5bkey:gene_model_track,CDSProductFeats:false%5d%5bkey:alignment_track,name:other%20alignments,annots:NG%20Alignments%7CRefseq%20Alignments%7CGnomon%20Alignments%7CUnnamed,shown:false%5d&v=205:299&appname=ncbiblast&link_loc=fromHSP) Next Match Previous Match

| Alignment statistics for match #1 | | | | |
| --- | --- | --- | --- | --- |
| **Score** | **Expect** | **Identities** | **Gaps** | **Strand** |
| 109 bits(120) | 8e-21 | 77/87(89%) | 2/87(2%) | Plus/Plus |

Query 1 GAGGTAATTGAATGTTTCTGCCCAGAGTTTCGACTGCTGTGGCTTGAAGCGAAATTTTGG

|||||||||||||||||| ||||||||||||| || ||||||||||||| |||||||||

Sbjct 209 GAGGTAATTGAATGTTTCGGCCCAGAGTTTCGGCTACTGTGGCTTGAAGTAAAATTTTGG

Query 61 AACGTCCTGCA--TGAGCGGGACAACA 85

||||||||||| | | |||||||||

Sbjct 269 AACGTCCTGCAATTTTGTGGGACAACA 295

**Alignment of target sequence from *O. volvulus* with *Wuchereria bancrofti***

Wuchereria bancrofti genome assembly W_bancrofti_Jakarta ,scaffold WBA_contig0001753

Sequence ID: [LM002423.1](https://www.ncbi.nlm.nih.gov/nucleotide/689544464?report=genbank&log$=nuclalign&blast_rank=31&RID=DURAUWV4016)Length: 8261Number of Matches: 1

Related Information

Range 1: 430 to 514[GenBank](https://www.ncbi.nlm.nih.gov/nucleotide/689544464?report=genbank&log$=nuclalign&blast_rank=31&RID=DURAUWV4016&from=430&to=514)[Graphics](https://www.ncbi.nlm.nih.gov/nuccore/689544464?report=graph&rid=DURAUWV4016%5b689544464%5d&tracks=%5bkey:sequence_track,name:Sequence,display_name:Sequence,id:STD1,category:Sequence,annots:Sequence,ShowLabel:true%5d%5bkey:gene_model_track,CDSProductFeats:false%5d%5bkey:alignment_track,name:other%20alignments,annots:NG%20Alignments%7CRefseq%20Alignments%7CGnomon%20Alignments%7CUnnamed,shown:false%5d&v=426:518&appname=ncbiblast&link_loc=fromHSP) Next Match Previous Match

| Alignment statistics for match #1 | | | | |
| --- | --- | --- | --- | --- |
| **Score** | **Expect** | **Identities** | **Gaps** | **Strand** |
| 105 bits(116) | 1e-19 | 75/85(88%) | 2/85(2%) | Plus/Plus |

Query 1 GAGGTAATTGAATGTTTCTGCCCAGAGTTTCGACTGCTGTGGCTTGAAGCGAAATTTTGG

|||||||||||||||||| ||||||||||| | || ||||||||||||| |||||||||

Sbjct 430 GAGGTAATTGAATGTTTCGGCCCAGAGTTTAGGCTACTGTGGCTTGAAGTAAAATTTTGG

Query 61 AACGTCCTGCATG--AGCGGGACAA 83

||||||||||||| | |||||||

Sbjct 490 AACGTCCTGCATGTTTGTGGGACAA 514

**Alignment of target sequence from *O. volvulus* with *Brugia malayi***

Brugia malayi (clone 8) 5S rRNA intergenic spacer DNA with two spliced leader sequences

Sequence ID: [L36060.1](https://www.ncbi.nlm.nih.gov/nucleotide/533165?report=genbank&log$=nuclalign&blast_rank=47&RID=DURAUWV4016)Length: 318Number of Matches: 2

Related Information

Range 1: 70 to 141[GenBank](https://www.ncbi.nlm.nih.gov/nucleotide/533165?report=genbank&log$=nuclalign&blast_rank=47&RID=DURAUWV4016&from=70&to=141)[Graphics](https://www.ncbi.nlm.nih.gov/nuccore/533165?report=graph&rid=DURAUWV4016%5b533165%5d&tracks=%5bkey:sequence_track,name:Sequence,display_name:Sequence,id:STD1,category:Sequence,annots:Sequence,ShowLabel:true%5d%5bkey:gene_model_track,CDSProductFeats:false%5d%5bkey:alignment_track,name:other%20alignments,annots:NG%20Alignments%7CRefseq%20Alignments%7CGnomon%20Alignments%7CUnnamed,shown:false%5d&v=67:144&appname=ncbiblast&link_loc=fromHSP) Next Match Previous Match

| Alignment statistics for match #1 | | | | |
| --- | --- | --- | --- | --- |
| **Score** | **Expect** | **Identities** | **Gaps** | **Strand** |
| 104 bits(114) | 3e-19 | 66/72(92%) | 0/72(0%) | Plus/Plus |

Query 1 GAGGTAATTGAATGTTTCTGCCCAGAGTTTCGACTGCTGTGGCTTGAAGCGAAATTTTGG

|||||||||||||||||| ||||||||||| | || ||||||||||||| |||||||||

Sbjct 70 GAGGTAATTGAATGTTTCGGCCCAGAGTTTAGGCTACTGTGGCTTGAAGTAAAATTTTGG

Query 61 AACGTCCTGCAT **72**

||||||||||||

Sbjct 130 AACGTCCTGCAT 141

**Alignment of target sequence from *O. volvulus* with *Mansonella streptocerca***

DEFINITION Mansonella streptocerca isolate ISCIII/Ms-1/2015 5S ribosomal RNA

gene region.

ACCESSION KT224442

VERSION KT224442.1

Sequence ID: Query_88733Length: 85Number of Matches: 1

Related Information

Range 1: 1 to 71[Graphics](https://www.ncbi.nlm.nih.gov/projects/sviewer/?RID=DUSKYYZS11N&id=lcl%7CQuery_88733&tracks=%5bkey:sequence_track,name:Sequence,display_name:Sequence,id:STD1,category:Sequence,annots:Sequence,ShowLabel:true%5d%5bkey:gene_model_track,CDSProductFeats:false%5d%5bkey:alignment_track,name:other%20alignments,annots:NG%20Alignments%7CRefseq%20Alignments%7CGnomon%20Alignments%7CUnnamed,shown:false%5d&v=0:74&appname=ncbiblast&link_loc=fromHSP) Next Match Previous Match

| Alignment statistics for match #1 | | | | |
| --- | --- | --- | --- | --- |
| **Score** | **Expect** | **Identities** | **Gaps** | **Strand** |
| 87.8 bits(96) | 1e-22 | 62/71(87%) | 0/71(0%) | Plus/Plus |

Query 234 GAGGTAATTGAATGTTTCGGCCCAGTTTTTCAGCTACTGTGGCTTGAAGTAAAATTTTGG

|||||||||||||||||| |||||| |||| || ||||||||||||| |||||||||

Sbjct 1 GAGGTAATTGAATGTTTCTGCCCAGAGTTTCGACTGCTGTGGCTTGAAGCGAAATTTTGG

Query 294 AACGTCCTACA 304

|||||||| ||

Sbjct 61 AACGTCCTGCA **71**

**Alignment of target sequence from *O. volvulus* with *Plasmodium falciparum***

RID

[DUSUUS5E11N](https://blast.ncbi.nlm.nih.gov/Blast.cgi?CMD=Get&RID=DUSUUS5E11N)

Query ID

[AF239766.1](https://www.ncbi.nlm.nih.gov/sites/entrez?cmd=Search&db=nucleotide&term=AF239766.1&dopt=GenBank)

Description

Plasmodium falciparum 5S ribosomal RNA gene, complete sequence

- No significant similarity found.
