# Supplementary figures and images for: O-5S quantitative real-time PCR: a new diagnostic tool for laboratory confirmation of human onchocerciasis
Source: Parasit Vectors. 2017 Oct 2;10:451. doi: 10.1186/s13071-017-2382-3 (PMC5625774; doi:10.1186/s13071-017-2382-3)

**Additional file 3: Figure S1**– Calibration curve of the novel O5-S qPCR


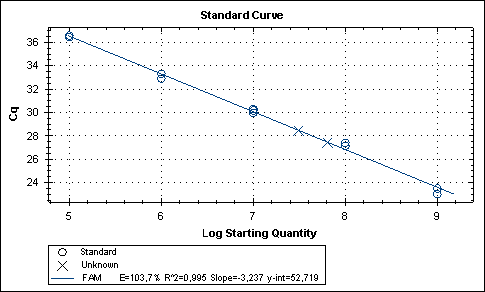

Supplement: Supplementary file 3 — Calibration curve of the novel O5-S qPCR. (DOCX 22 kb) [file 13071_2017_2382_MOESM3_ESM.docx]
