# Supplementary material for: O-5S quantitative real-time PCR: a new diagnostic tool for laboratory confirmation of human onchocerciasis
Source: Parasit Vectors. 2017 Oct 2;10:451. doi: 10.1186/s13071-017-2382-3 (PMC5625774; doi:10.1186/s13071-017-2382-3)
Supplement: Supplementary file 4 — XY-graph showing the correlation between O-150 qPCR and O-5S qPCR. (DOCX 19 kb) [file 13071_2017_2382_MOESM4_ESM.docx]

**Additional file 4 Figure S2**. XY-graph showing the correlation between O-150 qPCR and O-5S qPCR.
